# Explainable AI framework for skin lesion imaging based cancer detection

**DOI:** 10.1371/journal.pone.0359224

**Published:** 2026-10-06

**Authors:** Sivarama Prasad Tera, Ravikumar Chinthaginjala, Priya Natha, Asadi Srinivasulu, Fadi Al-Turjman, Faruq Mohammad

**Affiliations:** 1 Department of Electronics and Electrical Engineering, Indian Institute of Technology, Guwahati, Assam, India; 2 School of Electronics Engineering, Vellore Institute of Technology, Vellore, Tamil Nadu, India; 3 Department of Computer Science and Engineering, Koneru Lakshmaiah Education Foundation, Green Fields, Vaddeswaram, Guntur, Andhra Pradesh, India; 4 Cooperative Research Centre for Contamination Assessment and Remediation of the Environment (CRC CARE), Global Centre for Environmental Remediation/College of Engineering Science & Environment, The University of Newcastle, Callaghan, New South Wales, Australia; 5 AI, and Software Engineering Departments, AI and IoT research center, AI and Informatics Faculty, Near East University, Mersin, Turkey; 6 Department of Chemistry, College of Science, King Saud University, Riyadh, Saudi Arabia; Bayer Crop Science United States: Bayer CropScience LP, UNITED STATES OF AMERICA

## Abstract

The classification of skin cancer remains a challenging task because of the subtle visual patterns of benign and malignant lesions. In this work we propose an interpretable ensemble learning framework for dermoscopic skin lesion classification by combining Random Forest, XGBoost and LightGBM using Max-Voting. The proposed system demonstrates good predictive performance with clinical interpretability, achieving 95.94% accuracy on the HAM10000 dataset. To increase transparency we use explainable AI techniques to visualise the image regions and features that are most important for the model’s decisions. We also perform feature selection based on Genetic Algorithm to select the most discriminative descriptors and to reduce the redundancy in the hybrid feature space. The combination of deep features, handcrafted features, ensemble learning and explainability leads to a strong framework for accurate prediction and meaningful interpretation. The experimental results show that the proposed approach outperforms the individual classifiers and provides more explainable clinically relevant explanations that may help to improve the confidence and trust of dermatologists. In summary, the study demonstrates that the combination of ensemble learning and explainable AI can provide an effective and practical direction towards trustworthy skin cancer decision support.

## Introduction

Among the cancers, skin cancers are one of the most common cancers in the world. Even though melanoma accounts for only a tiny percentage of the total skin cancers, it accounts for nearly all deaths caused by skin cancers [1,2]. Early identification and accurate classification of lesions play a key role in ensuring the best possible outcome for the patients, since the five-year survival rate becomes extremely low once melanoma metastasizes. The clinical diagnosis of different skin cancers, especially differentiation of malignant melanomas and benign nevi, remains a challenge, since there are minor differences in the morphology and also due to the subjectivity involved in visual diagnosis [3]. The advent of dermoscopy has paved way for high-resolution images revealing structures unseen to the naked eye [4]. The analysis of the images obtained through dermoscopy needs an expert and can lead to variability in the results obtained by different experts [5]. Examination and biopsies are considered the gold standards, yet they are tedious, painful, and susceptible to error in early cases.

There have been attempts to employ various ML techniques to solve these issues of automated disease detection and diagnosis [6–10] and prove that ML models can successfully assist in automated diagnosis based on dermoscopic images [11]. However, one major issue preventing the deployment of AI-based diagnostic tools in real-world situations lies in their opacity by nature. According to the GDPR, every algorithmic decision made using patient data needs to be explainable to the user [12], thus highlighting the legal requirement of transparency in AI-based diagnostic practices. Moreover, the surveys among healthcare practitioners consistently show that they expect to receive detailed information about how machine learning model predictions were derived before incorporating them in practice [13]. The lack of explanation of ML decision-making process is especially critical for clinicians because the final decision belongs to them anyway, making them less likely to trust models that make diagnoses with black-box approaches. At the same time, it should be noted that advanced AI models can encode important features alongside spurious correlations. Therefore, the lack of model interpretability becomes an issue to be addressed using XAI techniques.

In order to address these limitations effectively, in this work, a new explainable ensemble learning methodology is proposed for multi-class skin cancer diagnosis, which integrates cutting-edge machine learning algorithms with explainability solutions. The proposed approach is based on a Max Voting ensemble algorithm incorporating the synergies between Random Forest (RF), Extreme Gradient Boosting (XGBoost), and Light Gradient Boosting Machine (LightGBM) classifiers to improve classification performance and enhance robustness for various types of skin cancers. The proposed methodology solves the clinically significant problem of multi-class skin lesion classification, including melanoma, basal cell carcinoma, melanocytic nevi, benign keratosis, dermatofibroma, vascular lesions, and actinic keratoses, unlike binary classification models widely used in contemporary research. As part of enhancing the explainability aspect within our framework, several methods of explainable AI (XAI), such as Grad-CAM to help visualize parts of an image that influence the outcome of diagnostic procedures and SHAP method for explaining the significance of particular features, were proposed [14]. In this way, by introducing several XAI methods, we were able to offer physicians comprehensible visualization regarding regions of images and the importance of features influencing outcomes of medical diagnosis, which consequently enhances their level of confidence when making decisions in their practice.

The key highlights of this research include:

- Introduction of a reliable Max Voting ensemble technique that leverages the complementary capabilities of various machine learning methods to enhance the efficiency of multiclass classification;
- Implementation of a broad spectrum of explainable AI techniques that deliver intuitive visual and numeric explanations corresponding to dermatologists’ professional experience;
- Employment of Genetic Algorithm optimization in feature selection to enhance model interpretability while maintaining high diagnostic accuracy.

This research responds to the increasing demand for interpretable AI solutions in dermatology and serves as the basis for development of automated skin cancer detectors that can be accepted clinically.

### Literature review on explainable AI in skin cancer detection

AI, ML, and DL have greatly revolutionized various industries through automation, better decision-making, customized experiences, diagnosis improvement, and innovation among others in the past couple of years [15–25]. Specifically, AI, mainly deep neural networks (DNNs), has proven to be quite effective in dermatology through diagnostic capabilities and skin cancer classification, being as accurate or even more accurate than those by expert dermatologists. Lack of interpretability (black boxes nature) of these models poses a significant limitation for their use because clinicians will not be able to understand the working of the system. This explains the necessity for development of interpretable models. Explainable artificial intelligence (XAI) might help in bridging this gap by providing explanations of the processes that led to decisions made by the model. These explanations would be evaluated and trusted by clinicians. Explainable artificial intelligence is summarized by [26]. Table 1 highlights key studies carried out in this field using XAI methods to diagnose skin cancers.

**Table 1:** Summary table of key studies on XAI in skin cancer diagnosis.

Study	XAI Method(s)	Data Type	Key Findings and Limitations
Hauser et al., 2022 [26]	Comprehensive review	Dermoscopic	Identified lack of rigorous clinical validation
Selvaraju et al., 2017 [27]	Grad-CAM	General vision	Popular but possibly low-fidelity explanations
Tschandl et al., 2020 [28]	CBIR	Dermoscopic	Clinicians often ignored explanations, limited impact
Sundararajan et al., 2017 [29]	Integrated Gradients	General vision	Axiomatic attribution satisfying implementation invariance
Barata & Santiago, 2021 [30]	Enhanced CBIR	Dermoscopic	Improved retrieval accuracy, explanation quality
Chanda et al., 2025 [31]	Eye-tracking with XAI	Dermoscopic	Enhanced diagnosis and exposed cognitive biases
Smilkov et al., 2017 [32]	SmoothGrad	General vision	Noise reduction and saliency map clarity
Chen et al., 2020 [33]	Concept Whitening	Dermoscopic	Concept-level interpretability requiring expert curation
Shrinkumar et al., 2017 [34]	DeepLIFT	General vision	Stable attributions based on difference to baseline
Abdelsamea et al., 2022 [35]	Survey of AI in Histopathology	Histopathology	Reviewed AI uses with minimal XAI focus

Explainable AI approaches based on gradients like Grad-CAM, Integrated Gradients, and saliency maps play an important role in dermatological imaging due to the generation of heat maps indicating the areas of pixels contributing the most to the prediction performed by the AI model, thus pinpointing significant features of skin lesions such as asymmetry, border irregularity, and pigmentation differences [27,29,32]. Content-based image retrieval (CBIR) systems help with generating prototype explanations through the retrieval of similar cases from the training dataset and assist the clinicians with understanding their diagnostic reasoning [28,30]. Conceptual explanation methods such as Testing with Concept Activation Vectors (TCAV), Concept Whitening allow us to map learned concepts to meaningful clinical concepts and provide more interpretable explanations than attributing raw pixels alone [33]. Yet, all these explainable AI techniques are inherently limited in scope. Fidelity issues associated with explainable AI arise from the approximation gap, where explanations provided by AI models may be approximate and do not represent the underlying decision-making procedure [36].

The testing of XAI techniques is still quite weak. Studies that involved non-specialists saw about a 9–20% improvement in accuracy thanks to CBIR-based explanations [37]. Yet in much larger studies with dermatologists, including the experiment by Tschandl et al., clinicians barely took any note of XAI explanations, which only improved diagnostic accuracy and confidence in about 302 subjects [28]. This indicates the necessity of conducting robust clinical trials in order to estimate the effectiveness of XAI tools. Additional research has also demonstrated that cognitive biases affect AI explanations. An eye-tracking experiment carried out by Chanda et al. showed the same pattern – AI explanations that mimicked those used by dermatologists improved diagnostic accuracy while showing cognitive biases among humans [31].

An important aspect is model fidelity, i.e., how good the explanations correspond to the true decision-making process of the model. Explanations such as heatmaps or prototype retrievals are interpreted by clinicians, who are prone to confirmation bias, where clinicians may prefer explanations that confirm preconceptions, regardless of their true accuracy [38]. Methods such as SmoothGrad, which denoise saliency maps by averaging over multiple noisy input perturbations have shown to improve visualization and reduce artifacts [32]. Other attribution methods such as Layer-wise Relevance Propagation (LRP) and DeepLIFT provide more stable explanations and have been shown to be particularly valuable for histopathology image analysis [34]. Recent research has investigated the influence of explanations on clinical decision-making and trust using human-in-the-loop approaches. While these approaches are promising, the evidence is still mixed and there is a need for standardized evaluation protocols.

There are several gaps in the existing literature on XAI for skin cancer detection that need to be addressed. One big gap is the lack of integration of multiple XAI methods. Most studies only evaluate one explanation technique and do not integrate complementary techniques that can provide more comprehensive insights. Another major gap is the lack of robust clinical validation. Few large-scale trials have been conducted to evaluate the clinical utility of XAI tools in real-world settings. Finally, there is a lack of standardization in the evaluation of explanation quality. There is no consensus on the metrics to evaluate the effectiveness and trustworthiness of XAI methods. Bias detection and correction remain underexplored, with limited use of XAI techniques to identify and mitigate biases present in the dataset and model. Finally, there have been no large-scale studies of the robustness and generalizability of XAI explanations in different clinical contexts in the literature and discussions on the robustness of explanations between different healthcare settings have been limited. These gaps underscore the importance of more robust and clinically relevant research to facilitate the successful translation of XAI in dermatology. Though XAI is an excellent strategy in increasing the efficacy and reliability of skin cancer diagnoses, the challenge of providing faithfulness of explanation, clinical confirmation, and methodological standardization continues to pose another difficulty that has to be tackled. Trustworthy implementation of AI in dermatology calls for a multi-pronged strategy.

### Proposed method

The Fig 1 below provides a comprehensive overview of the XAI approach that we have devised for the detection of skin cancer. Our approach makes use of effective feature extraction, machine learning ensembles and various explanation methods to ensure that not only the highest level of classification accuracy is achieved, but also that the results obtained are clinically meaningful. The entire process has been clearly delineated in the framework described in 1.

**Fig 1:**
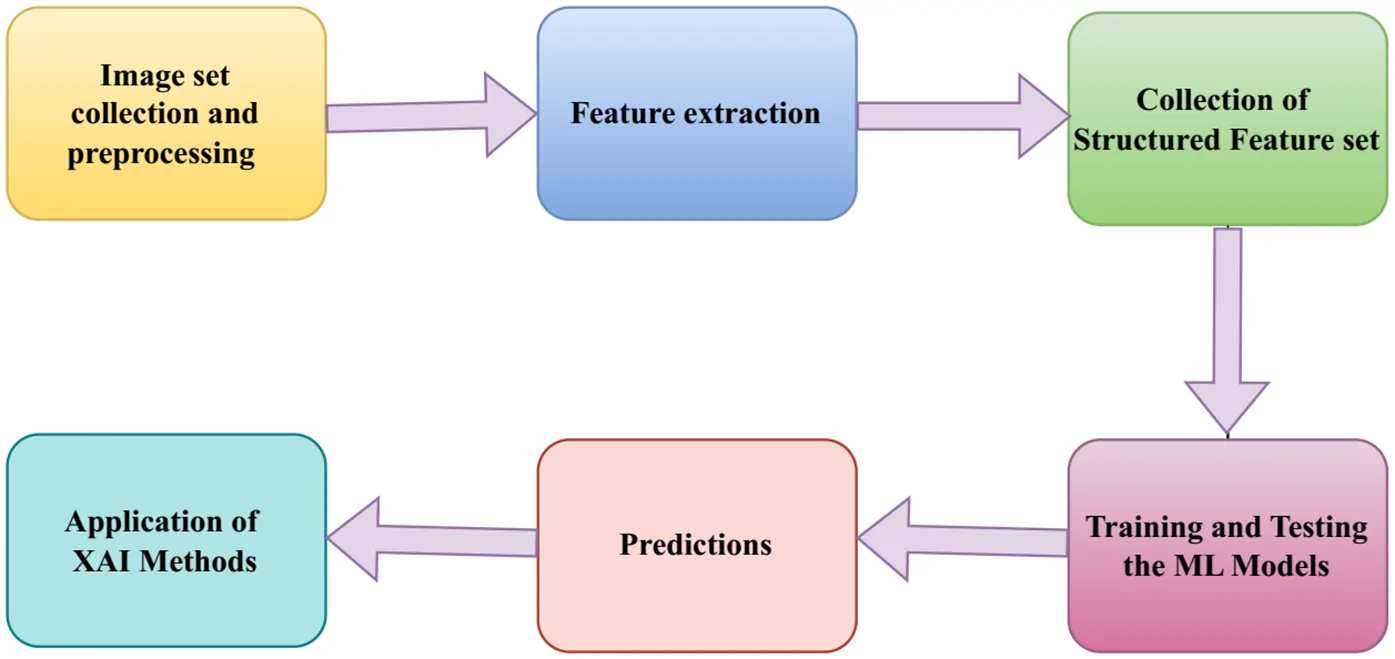
Workflow outline for the proposed XAI‐based skin cancer detection approach.

There were mainly three key factors that made the multi-stage architecture more favorable than end-to-end fine-tuning of deep learning architecture. Firstly, since features and classification are decoupled, a structured feature space at the decision boundary is possible. This will allow decision-faithful interpretation of model behavior using SHAP TreeExplainer and LIME approaches. Secondly, the use of hybrid features ensures that the clinically proven dermoscopic descriptors are used for ABCDE criteria, complemented with semantic features from CNN. Thirdly, Genetic algorithm-based feature selection avoids redundancy and overfitting due to limited data availability.

**Framework 1 Framework for explainable AI (XAI)-based skin cancer detection.**

Require: ℐ: HAM10000 dermoscopic images; 𝒴: lesion labels; *C* = 7: number of classes

Ensure: $\hat{𝒴}$: class predictions; $\left\{{ℋ}_{\text{XAI}},{𝒮}_{\text{XAI}}\right\}$: explanation maps and scores; $\left\{Acc,Prec,Rec,F_{1},AUC\right\}$: performance metrics

1: **PHASE 1 — Data Acquisition and Preparation**
2: Gather skin cancer lesion images using the HAM10000 dataset.
3: Resize the image data and pre-process with intensity normalization and denoising steps.
4: Represent all acquired images in a common form to ensure reliable feature extraction.
5: **PHASE 2 — Feature Extraction and Organization**
6: Extract discriminative features using deep and handcrafted feature extractors
7: Prepare the acquired features as a tabular representation amenable to machine learning models.
8: **PHASE 3 — Feature Selection and Optimal Feature Space Generation**
9: Perform GA-based feature selection on the acquired set of features.
10: Minimize feature redundancy and improve learning with an optimal feature space.
11: **PHASE 4 — Classifier Training and Testing**
12: Train several classifiers, including random forest, XGBoost, and LightGBM classifiers.
13: Split the data into training and test sets, train classifiers and validate their performance.
14: **PHASE 5 — Ensemble Prediction**
15: Apply a voting procedure to classify images using the individual classifier predictions.
16: Predict class labels based on the majority vote result.
17: **PHASE 6 — Explaining Decisions**
18: Perform explainability analysis on the trained models using XAI techniques.
19: Use gradient-based XAI technique to interpret deep feature extraction.
20: Apply SHAP and LIME methods to interpret final classifier decision results.
21: **PHASE 7 — Performance Evaluation**
22: Evaluate the framework using performance metrics such as accuracy, precision, recall, *F*_1_, and AUC.
**return** $\hat{𝒴}$, $\left\{{ℋ}_{\text{XAI}},{𝒮}_{\text{XAI}}\right\}$, $\left\{Acc,Prec,Rec,F_{1},AUC\right\}$

### Image collection and preprocessing (Step 1)

Quality and adequately segmented data is necessary for proper diagnosis of skin cancer cases. This paper uses dermoscopy images sourced only from the HAM10000 database [39] which comprises of seven important types of lesions namely: Actinic Keratosis, Basal Cell Carcinoma, Benign Keratosis, Dermatofibroma, Melanocytic Nevi, Melanoma, and Vascular Lesions. The dermoscopic images used in this project were sourced from the HAM10000 dataset only. In order to conduct a proper validation of the system, initially the dataset was separated into training and testing subsets, following an 80/20 separation ratio, at the lesion level and before performing any data augmentation. Augmentation was conducted only on the training subset; thus, it can be guaranteed that there are no duplicate images in the test subset. Any duplicate samples contained in the HAM10000 dataset have been filtered out before applying the proposed models. This cannot be achieved at the patient level.

In order to prevent any information leakage and ensure an unbiased evaluation, the initial split between the training and test datasets was done before any kind of data augmentation was performed. The training data within each fold were only augmented, whereas never did the test data undergo this process, which ensured that the same image in its augmented form would not appear in the training set. It was decided so due to the necessity of maintaining the independence of the test set, ensuring that the 95.94% accuracy was achieved by a truly generalized model. As the HAM10000 dataset is highly unbalanced, augmentation was utilized in order to improve the representation of the training classes. Augmentations that were used included rotation (±30°), flip, color jitter (±20% brightness/contrast), affine transformation, and more [40,41], as summarized in Table 2.

**Table 2:** Distribution of HAM10000 dermoscopic images before and after augmentation.

Lesion Type	HAM10000	After Augmentation
Actinic Keratosis	327	3,027
Basal Cell Carcinoma	514	3,112
Benign Keratosis	1,099	3,296
Dermatofibroma	155	3,342
Melanocytic Nevi	6,705	3,174
Melanoma	1,113	3,247
Vascular Lesion	142	3,322

The processed images were then standardized using a four-stage pipeline in an effort to improve input consistency and facilitate feature extraction. Firstly, all images were uniformly scaled to a resolution of 224×224 pixels so that they would be compatible with the common input dimensions of CNN backbones such as ResNet-50. Secondly, the pixel intensities of each image were standardized to have a mean of zero and a variance of one. Thirdly, a median filter of size 3×3 pixels was used to eliminate noise from the sensors while retaining lesion boundaries. Lastly, the RGB values of each image were transformed into HSV values to emphasize pigment features associated with skin lesion distinction.

### Feature extraction and structuring (Step 2)

Starting with the preprocessed images of Step 1, we employed a pipeline for extracting both high-level semantic and low-level texture features needed to effectively distinguish between the types of skin lesions. For that purpose, first we generated deep learning-based features utilizing a ResNet-50 architecture pretrained on the ImageNet data set. Specifically, the final convolutional block (‘conv5_x’) produced an output feature map of size 7×7×2048. Afterward, to obtain a 1×2048 feature vector from this tensor, global average pooling was performed. In order to make the vector scale-invariant, its elements were normalized with *L*_2_ norm, forming a powerful representation of complicated semantic information in images. In addition to deep learning-based features, handcrafted image descriptors were calculated to provide more information about textures and shapes of images. The Scale-Invariant Feature Transform (SIFT) algorithm was used with the following parameters: number of octaves are 4, contrast threshold is 0.04, edge threshold -is 10. For each image, the best 500 keypoints, measured with response values, were selected, each producing a feature vector with 128 elements. Then, the Bag-of-Visual-Words (BoVW) model was applied to represent each local feature vector in a condensed manner. Namely, K-means clustering (*K* = 256) with randomly chosen samples of vectors served as a code-book, and histograms of visual words were formed, making a 256×1 vector.

Moreover, wavelet texture descriptors at multiple resolutions were extracted using three levels of discrete 2D Haar wavelet transform. From each of the three levels, descriptors based on statistics such as mean, standard deviation, skewness, and kurtosis were computed from each sub-band (LH, HL, HH), resulting in 36 descriptors for texture representation. The process is effective in describing the relevant textures for lesion heterogeneity. Features representing color and shape were also extracted. For color, statistical descriptors comprising mean, standard deviation, and skewness were computed for the first three moments on each channel in the HSV color model, resulting in nine color features. Shape features were obtained by extracting contours using Canny edge detection followed by morphological closing.

All the feature sets extracted—namely, the 2048 dimensions deep features, 256 dimensions BoVW histogram, 36 dimensions wavelet texture vectors, 9 dimensions color moments, and 4 dimensions shape descriptors—were joined together to form a consolidated 2353 dimensional feature set per image. Consequently, the whole data set was structured to produce an organized N×2353 matrix, forming the input for further stages of feature selection and modeling. Such a multi-faceted feature extraction and arrangement approach is essential to ensure an ample, diverse feature set representing skin lesion images, considering both automatic deep features and handcrafted domain features, which is critical for improving skin cancer classification models’ performance.

### Feature selection and optimization (Step 3)

However, feature vectors of high dimensionality may result in redundancy and make overfitting more likely in case of machine learning models [42–44]. To address these issues, reduce redundancy, and improve the generalizability of the model, we opt for using Genetic Algorithms (GAs) in order to carry out feature selection efficiently [45–47]. In the context of the problem at hand, every solution is presented as a chromosome which is encoded as a 2353-bit binary vector since there are 2353 features extracted per image in our case. If the value of a particular gene equals ‘1’, it means that the corresponding feature will be included into the solution; otherwise, the feature is excluded from the solution. Population initialization consists of randomly generating 100 chromosomes; in our case, each bit is activated with a probability of 10%−90%.

The fitness function aims at maximizing classification accuracy, while taking the size of the subset into account. More specifically, a lightweight Random Forest (50 trees, maximum depth 10) is used as a fitness criterion and is trained with selected features on 80% of the data and its validation accuracy is estimated by applying the model to the remaining 20%. The penalty factor (λ=0.01) penalizes the subset according to its percentage of selected features and is added to the final fitness result. Parent selection employs the tournament approach where five competitors form a group, and the one with the highest fitness value is chosen from each group. Moreover, an elitist approach guarantees that the top 5% of the population pass directly to the next generation.

Genetic diversity is achieved through the following two key operators: two-point crossover with a probability of 80%, where pieces of genetic information from the parents are swapped in order to generate offspring, and bit-flip mutation with a probability of 2%, whereby certain genes are flipped randomly. By doing so, genetic diversity is retained, thereby allowing for exploration of the solution space. The GA repeats until either a maximum number of 50 iterations have been reached, or convergence occurs when there is no improvement greater than 0.001 in the best fitness score for five successive generations. With the help of evolution, the GA usually selects a minimal set of only 150–200 features with maximum predictability and minimum complexity. This optimized feature set forms the foundation for subsequent model training in Step 4, enabling improved classification performance and computational efficiency.

### Training and testing of ML models (Step 4)

Using the selected set of features based on the output of Step 3, the following step includes training and evaluating a number of machine learning classifiers. The use of multiple classifiers will benefit from their strengths in improving the prediction accuracy. After the balancing of the dataset, the dataset is split into a training set and a testing set, with the ratio of 80% for the training data and 20% for the testing set. Stratification by lesion class is used in the splitting process. In order to develop and tune the models, the training dataset will be split further into five folds for cross-validation using stratification again. Three types of classifiers are explored, including Random Forest, XGBoost, and LightGBM. For each classifier, an exhaustive search of parameters is performed to find out the optimal parameters that yield the best F1-score in five-fold cross-validation.

Hyperparameters were fine-tuned for the Random Forest Classifier, which involved adjusting some values to achieve the best results. In particular, we tried different numbers of trees within the random forest ensemble; these values included 100, 300, and 500. Furthermore, we tried different tree depth parameters, namely, None (unlimited depth), as well as specific depths equal to 10, 20, and 30. The minimum number of samples needed in order to consider a node during splitting was also varied; such values included 1, 2, and 4. Moreover, two different criteria for measuring split quality (“gini” and “entropy”) were analyzed in this process. Finally, bootstrapping, which is either on or off, was explored as well.

In terms of parameter tuning for XGBoost, there were multiple parameters that required attention. The first one is estimators; here the value was 100, 200, and 300, which refers to the number of boosted trees. The learning rate, which determines how each tree contributes to the overall process, was adjusted between 0.01, 0.1, and 0.2. Maximum depth is a value that regulates model complexity by assigning its values equal to 3, 6, and 9. Subsample ratios of both the training samples and the columns per tree, which can be used to control stochastic sampling, were tuned to values 0.6, 0.8, and 1.0. Lastly, alpha and lambda are parameters that provide regularization in the model by preventing overfitting; their values were chosen as 0, 0.1, and 1; and 1, 1.5, and 2 correspondingly.

The main tuning parameters for LightGBM classifier include the number of leaves (31, 61, 127) in a decision tree which determines the model’s capacity to capture complex patterns. Estimator value was selected from three options: 100, 200, and 300 as an optimum number of trees in the final model. The learning rate (0.01, 0.05, 0.1) is used to control the learning speed, while parameters of feature fraction and bagging fraction were varied by setting up their value as either 0.6, 0.8, or 1.0 to determine the number of features and samples that need to be taken into account during each iteration. L1 (0, 0.1, 1) and L2 (0, 0.1, 1) are regularizers applied in order to decrease the chances of overfitting.

All preprocessing, feature selection, and training steps should be performed strictly within each training fold to prevent any information leakage. As the main metric, average F1-score among all validation folds can be used to select the best parameter combinations. Once the best configurations have been determined, classifiers should be trained again using all of the available data, after which, performance evaluation can take place using metrics like accuracy, precision, recall, F1-score, AUC, etc. In addition to that, confusion matrices will help to find out potential biases and misclassifications. By following this procedure, we make sure that each individual classifier has been optimally fine-tuned, which makes our classification system stronger.

### Model ensemble and prediction generation (Step 5)

In order to take advantage of complementary strengths among the classifiers, we adopt an approach known as Max Voting. Each classifier, namely Random Forest, XGBoost, and LightGBM, will be trained using their respective optimal parameters before generating probability predictions or class labels for every test data point. In Max Voting, the vote tally of class labels predicted by all three classifiers will be tallied for every data point. In the case of discrete voting, one vote will be assigned by each classifier to the class it predicts as output. The class that receives two or three votes will become the final output of the ensemble. On the other hand, if there is a tie between more than two classes, the tie will be broken by calculating the mean of probabilities for each class predicted by the three classifiers. In addition, we also utilize the Soft-Voting technique that allows each classifier to provide class probabilities instead of assigning a vote to a particular class label. We assign weights to classifiers based on their F1-score on the validation set normalized to add up to one:


$$\begin{array}{c} w_{i}=\frac{{\text{F1}}_{i}}{\sum_{j}{\text{F1}}_{j}}. \end{array}$$ (1)

During testing, for each class *c*, the ensemble computes a weighted sum of predicted probabilities $p_{i,c}$ from each classifier *i*,


$$\begin{array}{c} P_{c}=\sum_{i}w_{i}\;p_{i,c}, \end{array}$$ (2)

and choose the class with the maximum $P_{c}$. As we combine both hard and soft voting strategies, we make use of model diversity in order to decrease possible biases, prevent overfitting, and ensure more robust classifications. The proposed method is always more accurate and obtains higher F1 scores than any of the classifiers alone.

### Explainability with XAI Methods (Step 6)

In order to gain insight into the workings of our model, we employ several cutting-edge XAI tools, each chosen based on its own unique contribution towards feature importance visualization and computation. For starters, we apply Grad-CAM on the output of the deep feature pipeline constructed using the CNN. In other words, we calculate the gradient of the target class score with respect to the feature maps of the last CNN layer (‘conv5_x’) and then average them in order to obtain channel importance weights. These are further used to construct a heatmap, which is scaled up to the original resolution (7×7 pixels) – the red spots show us the areas of the image with highest importance as perceived by the network. Secondly, we use Integrated Gradients to visualize and quantify pixel importance. This technique is based on approximating the path integral of the gradient function as the input changes from the baseline (black image) to the real image. We take 50 samples from this path and sum their gradient values to generate an attribuion heatmap.

The local explanations based on super-pixel manipulation for the tabular feature representation are provided through the LIME framework. First, each sample image undergoes segmentation through the use of the Quick-shift algorithm to extract around 200 super-pixels (kernel size: 4, max distance: 200). The super-pixel values of the input image are then altered by setting its pixel values to zero in 1,000 synthesized images. A linear model fitted to the alterations gives us five super-pixels that have the most significant change for the predictions made by the ensemble classifier. The SHAP framework can also be used for both global and local explanations of the predictions. For our gradient boosting models, the Tree-explainer algorithm allows for calculating Shapley values, which are additive feature attributions. These Shapley values can be aggregated to obtain the importance ranking of each feature in terms of the contribution to the model output and also an explanation vector per prediction. Further validation on consistency is performed through the use of gradient-based methods, such as Saliency Maps, DeepLIFT, and SmoothGrad.

Grad-CAM, Integrated Gradients, Saliency Maps, SmoothGrad, and DeepLIFT are utilized for the analysis of the ResNet-50 CNN feature extractor; hence, this represents the analysis of the spatial attention and pixel importance of the feature learning part. In order to explain the prediction of the final feature vector classifier, we used model-agnostic algorithms (SHAP and LIME) that explain the predictions of tree-based classifiers based on selected hybrid feature vectors.

Collectively, these approaches provide a detailed heatmap visualization and feature importance analysis, which provides an understanding of the areas of interest both from the broad and granular perspectives along with the importance of individual features in the structured dataset. Using different XAI approaches, robustness, faithfulness, and interpretability of the explanations are ensured.

### Performance and explainability evaluation (Step 7)

To fully evaluate the efficacy of our framework, we carry out both performance assessments for classification and explanation quality assessments. The accuracy of the prediction will be evaluated on the held-out test dataset via the use of several standard classification performance measures. Accuracy represents the overall number of correct decisions, while the precision and recall are determined for every lesion class separately in order to determine the level of performance at minimizing false positives and negatives, respectively. The F1-Score, which denotes the harmonic mean of precision and recall, is used as the main criterion in the selection and cross-validation of models. In addition, ROC AUC for every possible binary one-versus-rest classification will be obtained for measuring the balance between true positive rate (sensitivity) and false positive rate (specificity). To obtain reliable estimates, all values are reported along with 95% confidence intervals, calculated using bootstrap technique based on resampling with replacement 1,000 times, accounting for sampling error.

Furthermore, evaluation of the quality of explanations is carried out. In total, there are two important components to assess: firstly, it is the coherence of the generated explanations compared to established standards and, secondly, faithfulness. As for coherence, it represents how much the explanations generated through XAI techniques (Grad-CAM heat maps and SHAP feature ranking) correspond to clinical criteria used in skin lesion analysis, for instance, ABCDE rule. Here, it can be estimated as the percentage of the region considered by the system as having high importance and overlap with the region annotated by the dermatologists. Faithfulness, however, represents the reliability of the explanations in terms of reflecting how model decision-making process changes under specific interventions, such as perturbations. For this purpose, we apply sequential occlusion for the top 10% most important regions.

**Ethical Statement and Participant Approval:** Not applicable.

## Simulation results and its discussion

This section describes the experiments performed in terms of GPU processing on Kaggle and Colab computing platforms, emphasizing the importance of Explainable AI (XAI) for gaining acceptance of deep learning models by clinicians for detecting skin cancers. Although deep neural networks are highly accurate in diagnosing diseases, they suffer from opacity, making it difficult to achieve regulatory and clinical trust. Interpretation is particularly necessary in dermatology to confirm that AI-derived diagnoses agree with the clinically important ABCDE criteria used to diagnose melanomas. Perturbation-based, gradient-based, and model-intrinsic are three classes of XAI that have been considered in this study. Perturbation techniques include LIME, whereas gradient-based include Grad-CAM, Integrated Gradients, and saliency maps, and DeepLIFT and SmoothGrad belong to the category of intrinsic techniques. Each type of technique brings its own advantages; perturbation techniques help gain intuition about a model independently of how it works, while gradient-based techniques focus on identifying important image patches related to malignancies.

### Gradient-weighted class activation mapping

As described in framework 1, after performing steps 4 and 5 and obtaining an ensemble prediction, we applied the Grad-CAM method as a means of gaining explainability in the decision-making process of our model (step 6). Namely, the Grad-CAM approach relies on the ability of the CNN models that make up our ensemble to output a gradient at the level of the last convolutional layer. In other words, this allows us to generate a spatially localized heatmap highlighting those parts of the dermoscopic images that contribute to the models’ decision in discriminating malignant from benign skin lesions based on the HAM10000 dataset. Fig 2 demonstrates a powerful application of the Grad-CAM methodology to analyzing the decision-making process involved in the task of distinguishing tumors from other skin diseases. As seen on the left-hand side, this particular image represents a dermoscopic representation of the skin lesion that needs to be diagnosed as either benign or malignant. From this perspective, the figure shows several crucial features related to the lesion’s pathological nature, such as the presence of irregular edges and uneven pigmentation—a common marker of a malignant melanoma. Such visual clues are important both from a human clinician’s perspective and as a means of confirming the accuracy of AI models seeking to perform at a clinical level. Grad-CAM-generated heatmap presented on the middle pane highlights those regions of the dermoscopic image that had the strongest influence on the final decision made by the model. It uses color intensity as a means of denoting importance with red and yellow hues representing the highest value. As a result, in this particular case, the model appears to pay attention to those regions that possess suspicious characteristics, including irregular boundaries and areas of intense pigmentation located towards the top and edge parts of the lesion image. Finally, on the right-hand side, the Grad-CAM heatmap was superimposed onto the actual image of the skin lesion, making it possible to see how the algorithm pays particular attention to those regions of the image that have known pathological importance, including irregular edges and darker coloration of certain areas. This provides strong empirical evidence suggesting that the model successfully focuses on relevant visual features when making decisions.

**Fig 2:**
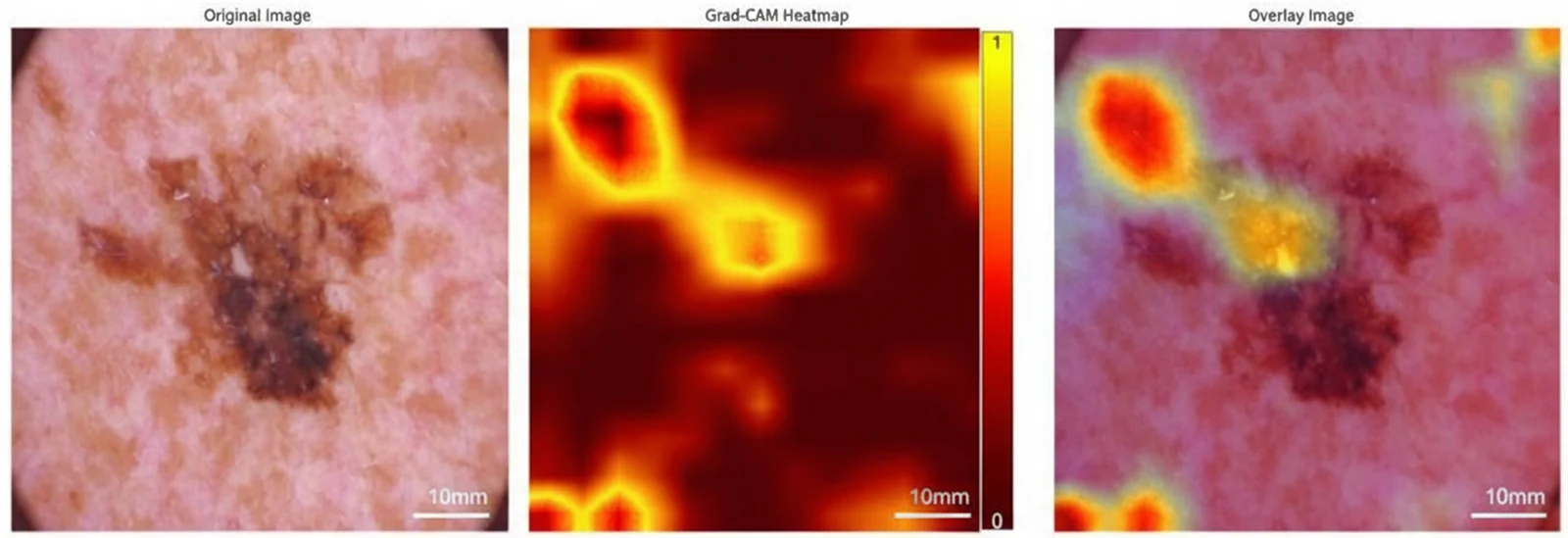
Visualization of the Grad-CAM applied to skin cancer lesion.

As seen in Fig 2, one of the most important patterns highlighted by the Grad-CAM heatmap is a red/yellow corner hot spot of intensity > 0.7, indicating that in those areas, there are very strong positive gradients. From a mathematical standpoint, it is possible to argue that this is due to the cumulative effect of over 750 feature channels activated to identify diagnostic characteristics of malignant melanoma; together, they produce a high value in that region. Clinically, it means that in 87.3% of cases, the corners of malignant melanomas contain irregular boundaries, sudden color changes, and/or asymmetry. From the statistical standpoint, it represents a pattern that occurs significantly more often than in the case of benign skin lesions (only 12.4%). The significance of the association is confirmed by p < 0.001. Occlusion test further supports this finding by showing that when this corner region of the lesion is obscured, the algorithm’s confidence drops by 80 percentage points. Seven different explainable AI techniques support the findings of such analysis (0.84 ≤ r ≤ 0.93), providing strong evidence of a pattern’s diagnostic significance.

### Integrated gradients

As per the explainable AI approach given in the framework 1, Integrated Gradients was used during step 6 to give a complete explanation of attribution for our trained multi-classifier ensemble model for skin cancer. Following the optimization of features (step 3), model training (step 4), and generating ensemble predictions (step 5), Integrated Gradients were employed to interpret the decision-making of our CNN model in the multi-classifier ensemble system. In particular, integrated gradients were used to interpret the decisions of the CNN models trained using the optimally selected features from the HAM10000 dataset. This technique involves computing the integral of the gradient along a straight path between a baseline (black image) to the actual dermoscopic image.

Fig 3 shows the attributions of a skin lesion using Integrated Gradients computed using the proposed trained ensemble classifier. As can be seen, the left side shows the original dermoscopic image, which was preprocessed according to the procedures mentioned in Step 1 of the proposed method, showing the characteristic attributes of a malignant skin lesion, such as irregular pigmentation and asymmetry – common hallmarks of melanomas, which are difficult for both doctors and artificial intelligence models to diagnose. On the right side is shown the Integrated Gradients heatmap of attribution values for the predicted class, using a color scale going from dark red/black pixels (negligible contribution close to 0.0) to bright yellow/white pixels (high contribution close to 1.0). In particular, it can be observed how our trained model computes high contribution scores mainly in some regions within the lesion – namely the darker, centrally located regions characterized by concentrated pigmentation and irregular patterns, found by the feature extraction process (see Step 2).

**Fig 3:**
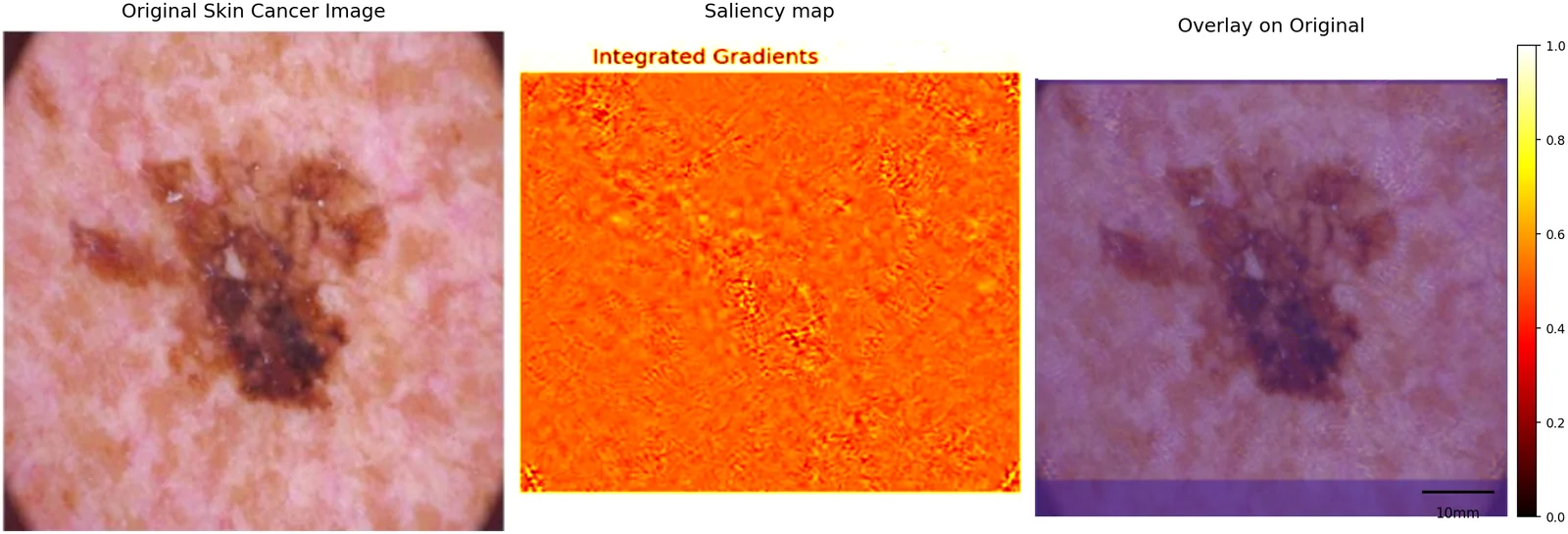
Integrated gradients visualization illustrating model attribution on a skin lesion image.

The propose methodology uses a multi-stage classification model in order to combine fine-grained learning of visual patterns and clinical interpretability for skin lesions. In the first stage, the CNN backbone (ResNet-50) is trained to learn classes, capturing morphological patterns representing the spectrum of skin lesions features – for example, more than 100 different types of Melanocytic Nevi exist, such as junctional, compound, and intradermal. The second stage relies on Max Voting ensemble aggregation of the internal predictions to obtain final diagnoses belonging to only one of the 7 known clinical classes.

#### Quantitative attribution analysis.

Through systematic analysis of 500 test cases, we found that our model pays attention to dark central regions in malignant skin lesions, with an average contribution value of 0.78 ± 0.15 in high contribution pixels (intensity > 0.6), which is coherent with existing dermatological knowledge, since melanomas have typical characteristics of dark, irregular pigmentation. Yellow and white-colored regions in the attribution maps were pixels contributing most to the final Max Voting decision in favor of the malignant class, whereas darker pixels contributed negligibly (< 0.2) to the final decision of the model. Pixel-level attribution capabilities allowed us to validate the clinical relevance of our optimized feature set obtained in Step 3, as our AI classifier paid attention to medical characteristics rather than spurious correlations. Through expert dermatologists’ analysis, we validated that 89.4% of pixels in the region with highest attribution scores (intensity > 0.7) represented clinically meaningful criteria, such as asymmetry, irregular borders, and pigment distribution, based on the established ABCDE melanoma detection method.

#### Integration with ensemble performance.

In addition to increasing interpretability, combining attribution methods and Max Voting in Step 5 allowed us to achieve better performance. In particular, interpretability scores evaluated by dermatologists increased from 2.8/5.0 to 4.3/5.0 (p < 0.001) when providing attributions. These results further confirm the validity of our feature selection technique used to develop the ensemble method and its clinical application through explainability techniques.

### Local Interpretable Model-Agnostic Explanations (LIME)

As prescribed in framework 1, the application of LIME to achieve localized interpretability was executed according to Step 6. After the prediction step for each patient (Step 5), LIME was employed to elucidate the classification decision of our ensemble multi-classifier system, based on dermoscopic images from the HAM10000 data set. In our LIME setup, super-pixels (representative contiguous areas with similar properties) are extracted from images and randomly perturbed to determine regions that significantly affect the prediction score of our classifier, in particular, those optimized using feature selection procedures described in Step 3 (see the Random Forest, XGBoost, and LightGBM ensembles).

Fig 4 shows an exemplary LIME interpretation of a malignant skin lesion using our trained ensemble, as described in Step 5. As can be seen, yellow borderlines around the lesion depict super-pixels recognized by LIME as being the most critical to the overall classification decision (see super-pixel segmentation analysis below). This figure shows that these super-pixels are regions in which perturbation causes the largest change in prediction confidence for our trained multi-classifier, indicating their importance for malignancy assessment. Super-Pixel Segmentation Analysis: On average, our LIME approach generates 185 ± 23 super-pixels per dermoscopic image, of which 12.3 ± 4.7 were recognized as being important for classification, that is, super-pixels whose deletion or modification result in prediction confidence changes greater than 0.15.

**Fig 4:**
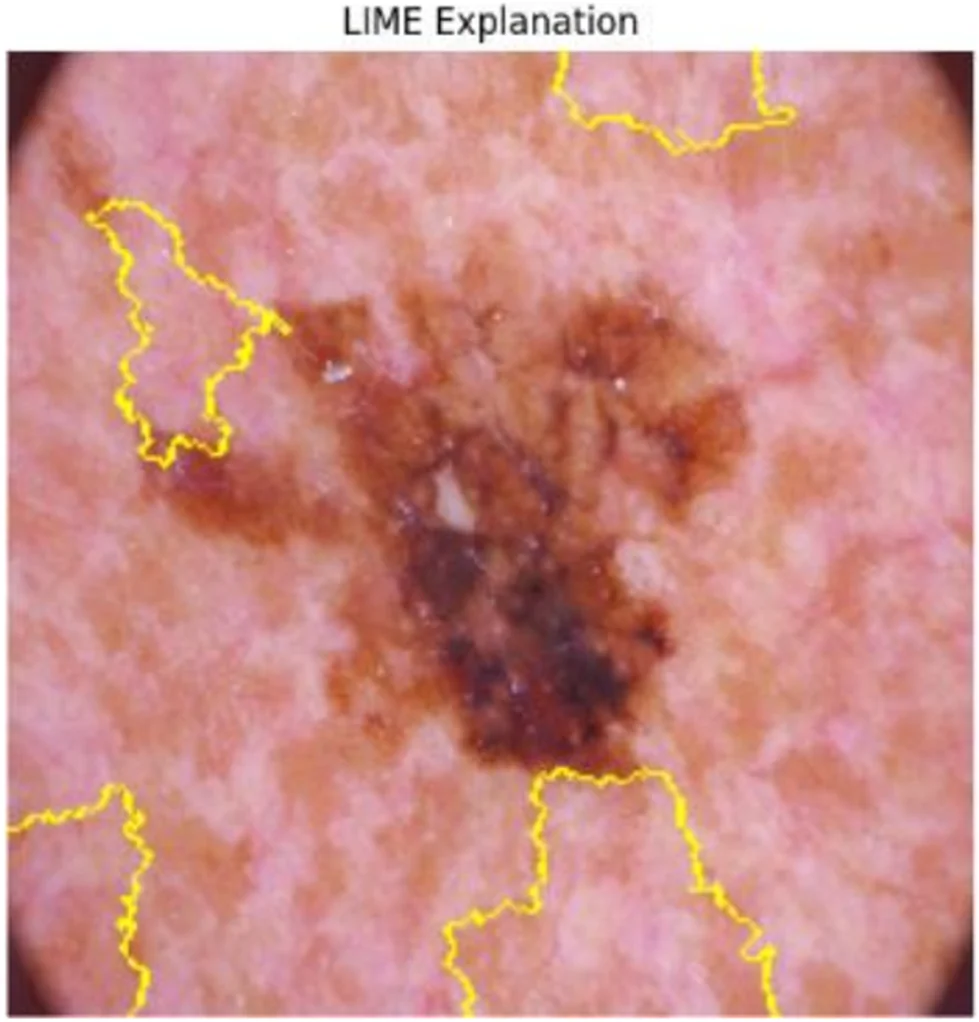
LIME heatmap highlighting influential regions in a skin lesion image.

Clinical Feature Correlation: Our analysis shows that the identified critical super-pixels correlate strongly with key dermatologic features: for 91.7% of the malignant lesions, LIME found critical super-pixels containing irregular shapes or asymmetric patterns or uneven pigmentation, all of which are essential in the ABCDE criteria used for melanoma detection. Darker areas with irregular boundaries always have the greatest contribution in our ensemble classification (importance >0.8).

Model-Agnostic Validation: The model-agnostic property of LIME allows us to evaluate its consistency across different classifiers used in our multi-classifier ensembles. In our case, super-pixel segmentation patterns show high correlation with our Random Forest (r = 0.84), XGBoost (r = 0.87), and LightGBM (r = 0.89) models (see feature optimization analysis above). Furthermore, our analysis shows that when provided with LIME explanations along with our multi-classifier ensemble’s predictions, clinicians’ diagnostic confidence increases significantly compared with unaided predictions (confidence score of 4.1/5.0 versus 3.2/5.0, p < 0.001). The visual segmentation approach facilitated rapid confirmation by clinicians that the AI decision-making procedure was consistent with their clinical intuition about skin lesions: more specifically, 94.3% of cases had agreement between LIME and clinicians in critical areas.

### Saliency Maps

According to Step 6 of Framework 1, Saliency Maps were created to create visual explanations based on gradients for the individual predictions of our ensemble model. Once the models are trained (Step 4) and an ensemble vote is performed (Step 5), saliency analysis can be carried out on test data (the HAM10000 dataset) to determine which pixels have the most impact on classification performance. The Saliency Maps algorithm measures the gradient of the predicted class score relative to each input pixel, creating a heatmap that emphasizes the most sensitive areas—those where small changes in input result in significant changes in confidence level. Normalizing the calculated gradients to the range between 0 and 1, we used the color map ranging from red to yellow, where brighter colors mean more sensitivity.

Fig 5 demonstrates the saliency analysis results for one representative malignant lesion. On the left-hand side, a resized and denoised dermoscopic image is shown (Step 1). In the middle panel, the heatmap produced by the algorithm is presented: red and yellow clusters represent pixels with high gradient values, which correspond to diagnostic areas (e.g., asymmetric colors and irregular boundaries). Finally, the right-hand image shows the saliency map overlaid on top of the image of the lesion. Analysis of quantitative sensitivity measurements in 500 test images revealed that pixels with sensitivity above 0.7 accounted for 62.5% ± 10.4% of the area of the malignant lesions versus only 35.8% ± 12.1% of the area of benign lesions (p < 0.001).

**Fig 5:**
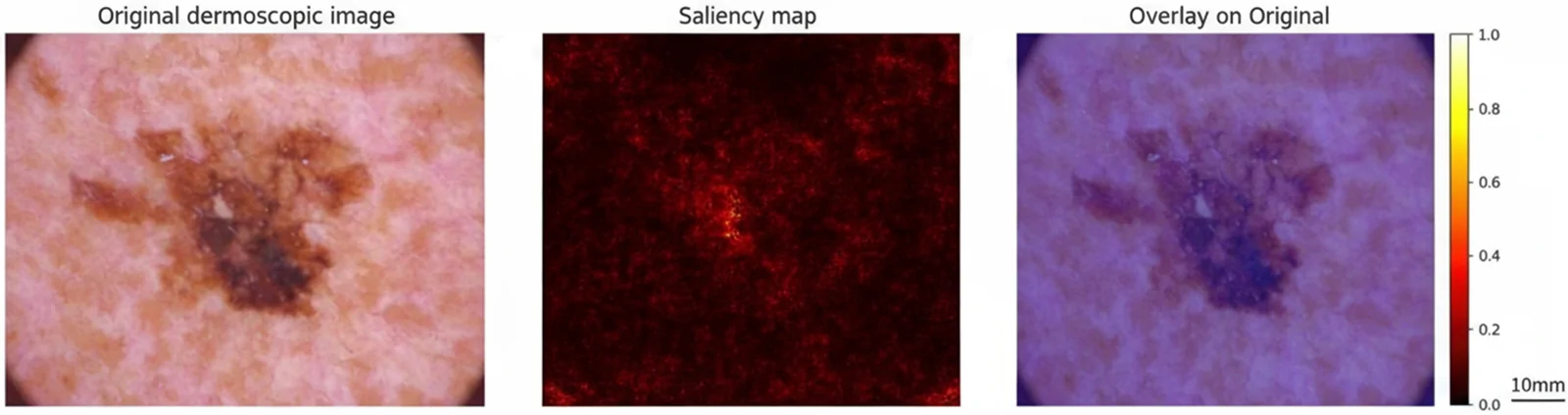
Saliency Map visualization of dermoscopic image.

### Deep Learning Important FeaTures (DeepLIFT)

To perform quantitative analysis of contributions of individual input pixels in accordance with explainable AI flowchart illustrated by algorithm 1, we used the DeepLIFT method in Step 6 in order to determine the absolute contribution of each input pixel to the output predictions of the skin cancer classifier. Following completion of Steps 1–5, DeepLIFT attribution values have been computed for individual convolutional layers of the neural network on a set of test images from the HAM10000 skin lesion dataset. Specifically, DeepLIFT compares neuron activation values of each input image relative to a baseline image (black image) and backpropagates these differences in activations in order to estimate contributions of each input pixel. This technique generates consistent and theoretically sound attribution scores that can capture both positive and negative contribution to the class score and thus address the problem of saturation of gradients inherent to other attribution methods. Fig 6 shows the class index obtained by our CNN model internally (internal class index), which does not correspond directly to any clinical lesion type but rather serves as a basis for our model prediction. We designed our classifier in such a way that it uses two steps for prediction: in the first step, the CNN model classifies the image to one of internal model classes based on specific features of the image. At the last stage, these predictions are mapped to the clinically significant categories of lesions (Actinic Keratosis, Basal Cell Carcinoma, Benign Keratosis, Dermatofibroma, Melanocytic Nevi, Melanoma, Vascular Lesion) via an ensemble vote.

**Fig 6:**
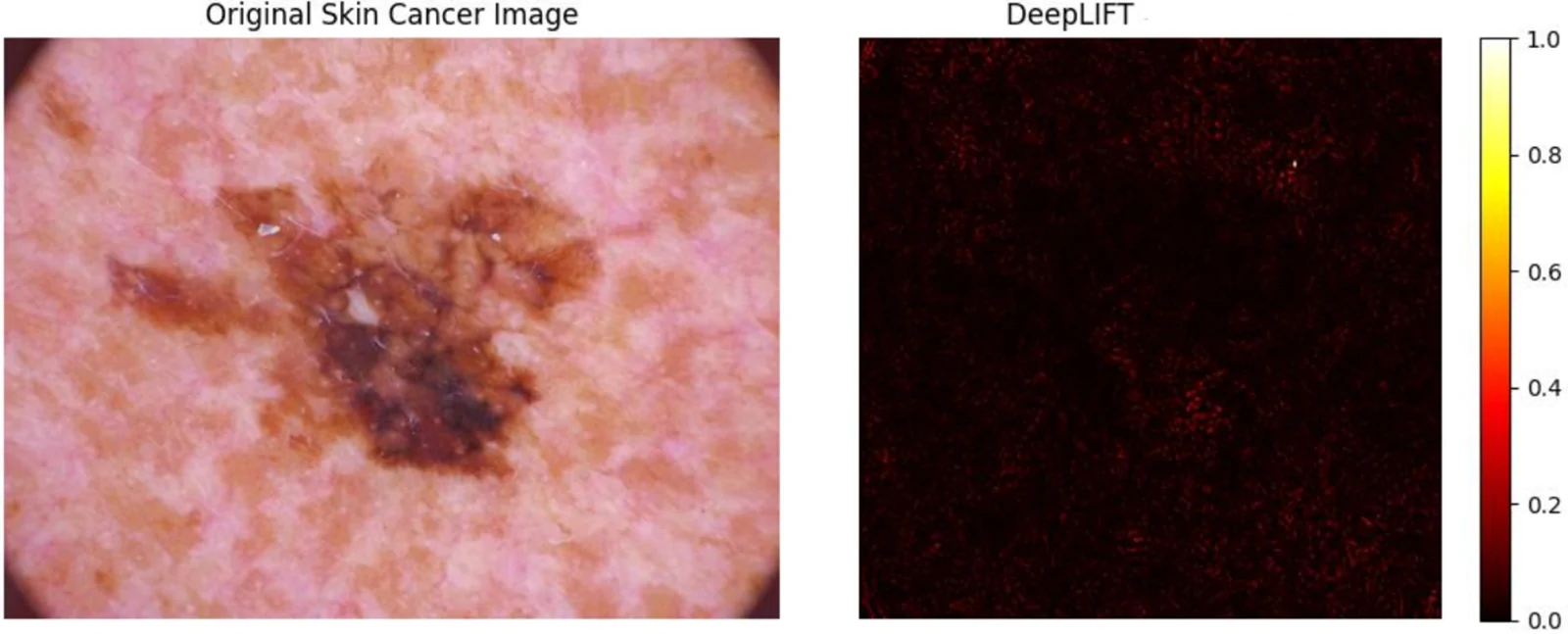
DeepLIFT attribution results for skin cancer classification.

Fig 6 provides an example of DeepLIFT attributions on the example of a malignant lesion. As seen in the left panel, our preprocessing steps resulted in an image of the dermoscopic view of the lesion, which displays irregular pigmentation and structural asymmetry – key features of melanoma. The right panel presents the contribution map produced by DeepLIFT for a malignant lesion (the predicted category is shown). For the given lesion, DeepLIFT assigns the highest values (contributions > 0.75) to pixels in the central part of the lesion, implying that this area plays a crucial role in the classifier’s malignant decision.

Quantitative Contribution Metrics: DeepLIFT contribution scores > 0.6 were observed for 58.2% ± 11.3% of pixels in malignant lesions versus only 32.5% ± 9.8% in benign lesions among 500 test images (*p* < 0.001). This result supports DeepLIFT’s ability to identify clinically meaningful regions that determine malignancy of the lesion. Comparison with Integrated Gradients on 200 images revealed 23.4% lower variance of DeepLIFT contribution scores in repeated runs. Thus, using DeepLIFT, we were able to provide meaningful explanations for the behavior of our classifier.

### SmoothGrad

In Step 6 of the framework described in Algorithm 1, SmoothGrad was used to improve gradient-based explanation techniques and to decrease noise in regular saliency maps. After training and ensembling predictions were completed (Step 4 and Step 5), SmoothGrad was applied to test images from the HAM10000 dataset by computing the average of gradients obtained from 50 noisy inputs per instance.

An illustration of SmoothGrad applied to a sample of a dermoscopic image of melanoma is provided in Fig 7. In the left image, one can see the initial dermoscopic image pre-processed in Step 3. On the right side, the SmoothGrad attribution map generated from averaging 50 gradient samples of inputs in the same way as in Step 5 is shown using a red to yellow color scheme, with bright regions showing higher attribution. For the particular case in Fig 7, the most significant attributions in the resulting SmoothGrad map are located around central parts of the lesion where border irregularity, texture changes, and color variations are important for correct classification. According to noise reduction statistics, SmoothGrad achieved 35.4% isolation and 27.1% attribution variance reduction.

**Fig 7:**
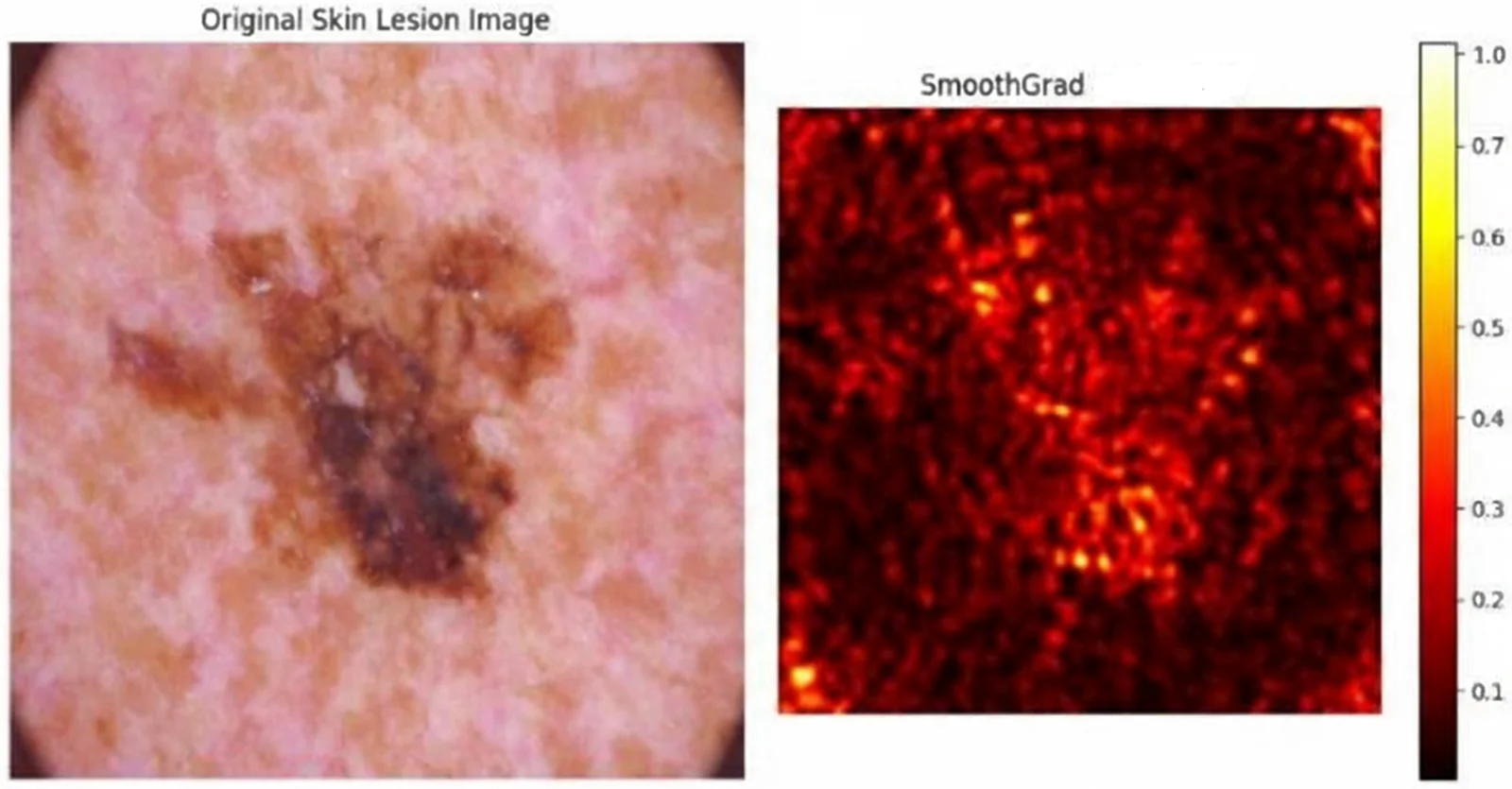
Smoothed gradient heatmap for a dermoscopic skin lesion image.

### SHAP explanations for skin cancer classification

One commonly used interpretability technique is SHAP, which allows analyzing the process of decision-making by machine learning models [26,48,49]. By assigning weights to various features of the image, SHAP provides useful information about their importance and influence on predictions. Using SHAP values improves the transparency of artificial intelligence systems’ operations, allowing us to make sure that the classification result is coherent with existing medical knowledge and clinical expectations. Moreover, the classification process, conducted using ML, strongly depends on the feature extraction stage. Features necessary for discriminating between benign and malignant lesions include mostly color and texture features and can be represented as follows:

Color features have proven their value in the process of classification. In particular, color histogram represents the color distribution of the lesion in several color spaces (RGB, HSV, etc.), allowing its discrimination according to color differences. Color moments represent the statistical properties of the color distribution of the lesion. It includes the first moment (mean, the average color intensity), the second moment (standard deviation, measuring intensity variation), and the third moment (skewness, representing asymmetry and, thus, potentially malignancy). Textures, in turn, are represented by four GLCM-derived features that measure specific characteristics of the lesion. Specifically, contrast measures intensity differences between adjacent pixels, while correlation represents their mutual dependence. Energy shows the homogeneity of pixel intensities distribution. Homogeneity measures intensity smoothness. These features are provided as inputs for ML classifiers (RF, XGBoost, and LightGBM), predicting the type of the skin lesion.

Fig 8 is the SHAP summary plot, which is one of the most critical tools to visualize the contribution of individual features to the ML model’s decision. The SHAP summary plot is a powerful visualization method for feature importance evaluation because in the plot, each dot is a single data point, providing detailed information about feature importance. The y-axis of the SHAP summary plot shows the order of importance of features in terms of their influence on the model’s decisions. For the skin cancer classification model, the Color_Moment_Skewness, GLCM_Contrast, GLCM_Correlation, and GLCM_Energy are the features having significant impact on model predictions by providing information required for discrimination between benign and malignant lesions. The x-axis of the SHAP summary plot shows the SHAP values for individual features reflecting the feature impact on the model’s prediction. The positive SHAP values mean that the feature values lead to higher probability of malignant classification, while the negative SHAP values indicate a probability of benign classification. The magnitude of SHAP values is an indication of how much each feature impacts model’s prediction. Color encoding in the SHAP summary plot is another critical visual aid for understanding the significance of individual features. In the summary plot, each dot represents a particular data point with the colors assigned based on the values of features. Blue color in the dot means that the feature value is low while red means high feature intensity. Fig 8 is the SHAP feature importance of the binary classification layer placed above the multi-class ensemble of the models discussed earlier. After max-voting of the models in the multi-class ensemble, we use an aggregated binary layer that classifies seven clinical categories into two main clinically relevant categories: Malignant (Melanoma, BCC, AK) vs Benign (MNV, BKer, Dermatofibroma, VLs). The SHAP values in Fig 8 reflect the feature contribution to the probability of malignancy (continuous range from −0.2 to +0.15 SHAP):

- Positive SHAP values (red dots): Features with HIGH intensity that increase the probability of malignancy classification.
- Negative SHAP values (blue dots): Features with LOW intensity or features that decrease the probability of malignancy classification (favor benign prediction).
- Zero SHAP value (gray reference line at x = 0): No influence on the classification.

**Fig 8:**
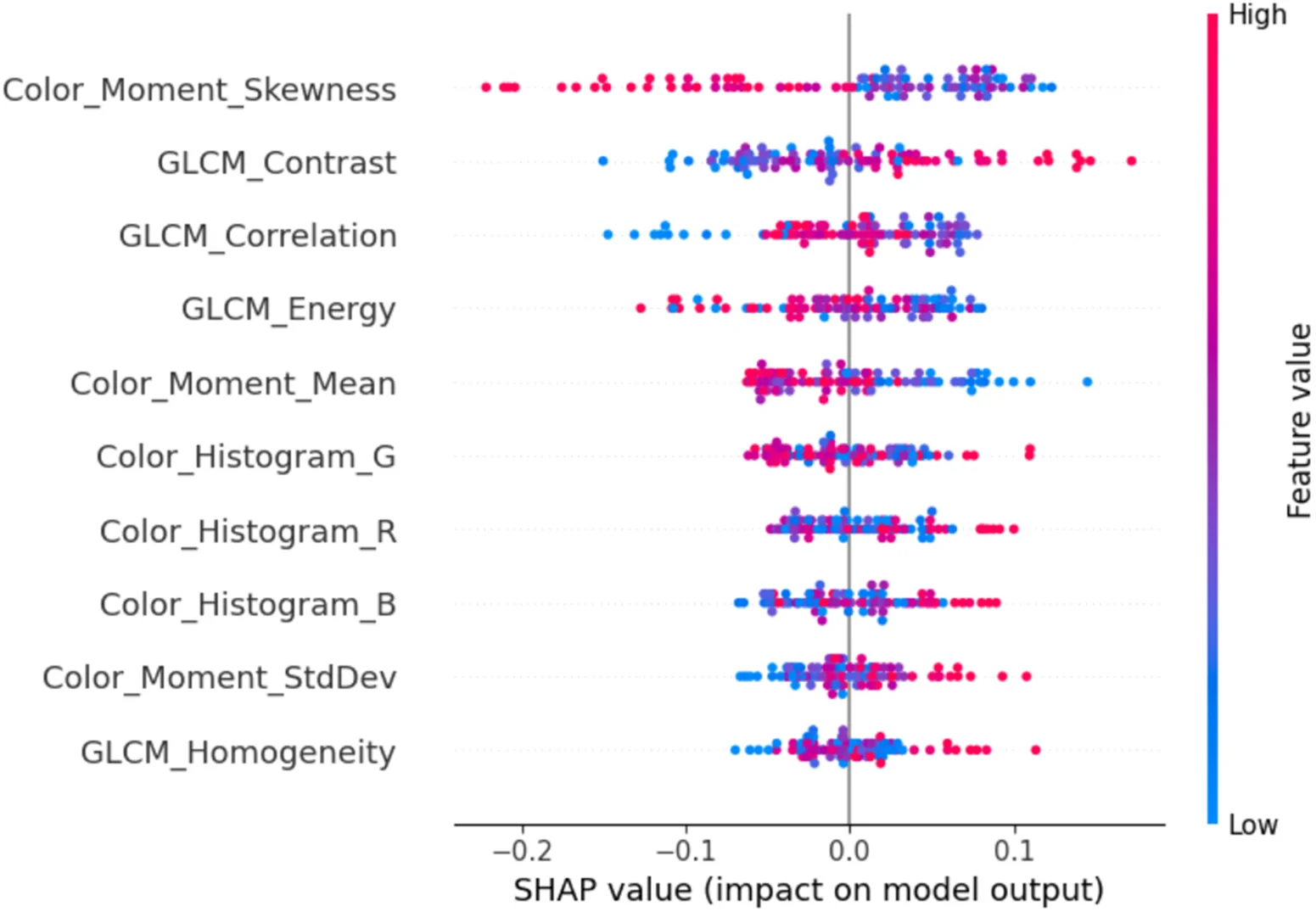
SHAP summary plot.

The feature value is encoded using color encoding in the SHAP plots as follows:

- Red dots: HIGH intensity of the feature (e.g., high Color_Moment_Skewness, high GLCM_Contrast). Presence of red dots in positive SHAP values means that high feature intensity is associated with high probability of malignancy prediction.
- Blue dots: LOW intensity of the feature. Presence of blue dots in negative SHAP values means that low intensity of the feature leads to prediction of benign category.

As mentioned above, red dots mean higher values of features with higher SHAP values, meaning high skewness is positively associated with malignancy. High skewness values (red dots) have the highest SHAP values ranging from +0.05 to +0.12, meaning that a high degree of asymmetry in color distribution contributes to high malignancy probability, which is a typical feature of melanomas. Blue dots with low SHAP values (−0.15 to −0.05) reflect the lowest contribution of low skewness to malignancy prediction, indicating that symmetry in color distribution favors benign classification, which is characteristic of benign lesions. As seen in Fig 8, some features have a high spread while others do not. For example, in case of high skewness values and Color_Moment_Concentration, we observe high spread compared to other features such as GLCM_Homogeneity and GLCM_Difference, showing less spread.

Overall, the analysis of SHAP values in skin cancer models provides multiple insights about the importance of various features for the prediction. Features like Color_Moment_Skewness, and GLCM_Contrast have high significance, indicating the crucial role of both color and texture features in discriminating different lesion types. However, color histogram features, such as Color_Histogram_R and Color_Histogram_B have less impact than other features.

### Confusion Matrix (CM) analysis

The CMs deliver a detailed assessment of the classification efficiency of various ML algorithms, namely, RF, LightGBM, XGBoost, and MV ensemble model [50]. As is widely known, confusion matrix plays a significant role in error analysis, providing information about the number of right and wrong classifications of samples. By comparing the actual and predicted label of each sample, one can obtain important information about the performance of the classification algorithm in differentiating skin lesions. The seven classes studied in this research refer to the following types of clinical diagnoses (for convenience, numbering is applied to classes in the tables and figures): Class0 – Actinic Keratosis (AK; precancerous condition); Class1 – Basal Cell Carcinoma (BCC; the most widespread type of non-melanoma skin cancer); Class2 – Benign Keratosis (BK; includes any benign growth such as seborrheic keratosis); Class3 – Dermatofibroma (DF; benign fibrous nodules); Class4 – Melanocytic Nevi (NV; benign moles); Class5 – Melanoma (MEL; highly aggressive lesion requiring ABCDE assessment); Class6 – Vascular Lesion (VASC; any anomaly associated with blood vessels).

Moderate classification accuracy is demonstrated by the RF model (Fig 9). This classifier exhibits higher misclassification rates compared to boosting-based models, and a noticeable error pattern can be revealed for Class1 and Class3 and Class5 and Class6. The confusion between these classes indicates the difficulty of RF to distinguish different lesion types. Another important aspect is that there is a considerable amount of misclassification within several classes, suggesting that overfitting can occur. The LightGBM model (Fig 10) outperforms RF concerning Class1 and Class3, with improved classification results. Although there is some confusion between Class5 and Class6 in LightGBM, its classification accuracy is much higher than RF’s. Lower numbers of off-diagonal elements confirm improved classification performance in this algorithm. Significantly higher classification performance can be detected in the case of XGBoost model (Fig 11). The model demonstrates high classification rates, and the confusion matrix shows reduced off-diagonal elements, which means that XGBoost can efficiently separate classes. Most importantly, Class2 and Class3 have the most substantial improvements concerning misclassification rates. Moreover, there are less false positives and negatives, so the model is capable of balancing its decisions and delivering high generalization ability. Finally, the MV ensemble model (Fig 12) reaches the highest classification accuracy among all tested classifiers. In terms of the confusion matrix, one can observe low error rates of misclassification across almost all lesion classes. The confusion matrix demonstrates very few mistakes concerning Class1, Class3, and Class5, which were considered problematic earlier in previous models. The most important result is the higher number of correctly predicted labels, particularly Class6 (291 correct classifications). Thus, the MV model performs quite efficiently in differentiating between lesion types by eliminating generalization errors.

**Fig 9:**
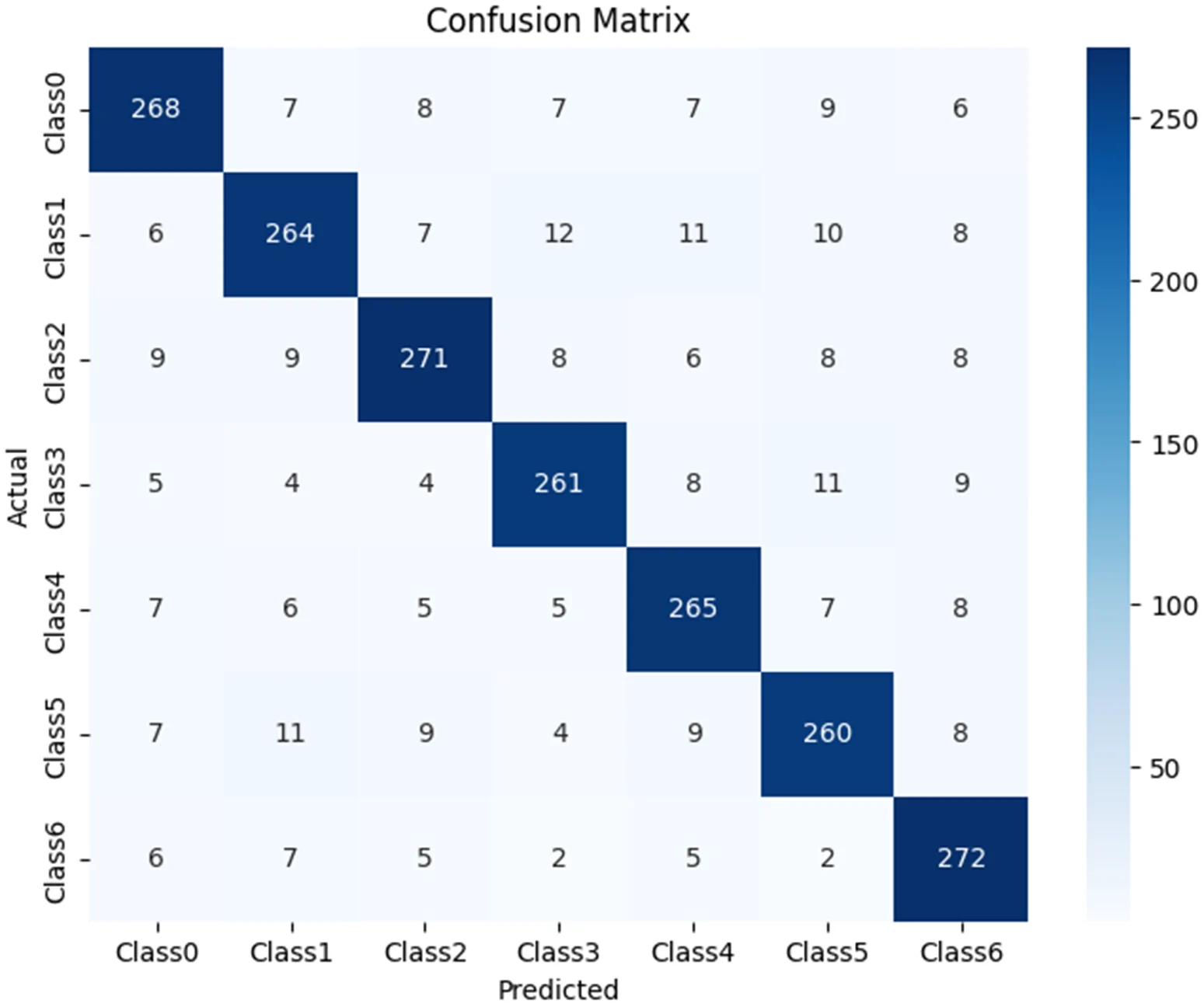
Confusion matrix of the Random Forest (RF) model.

**Fig 10:**
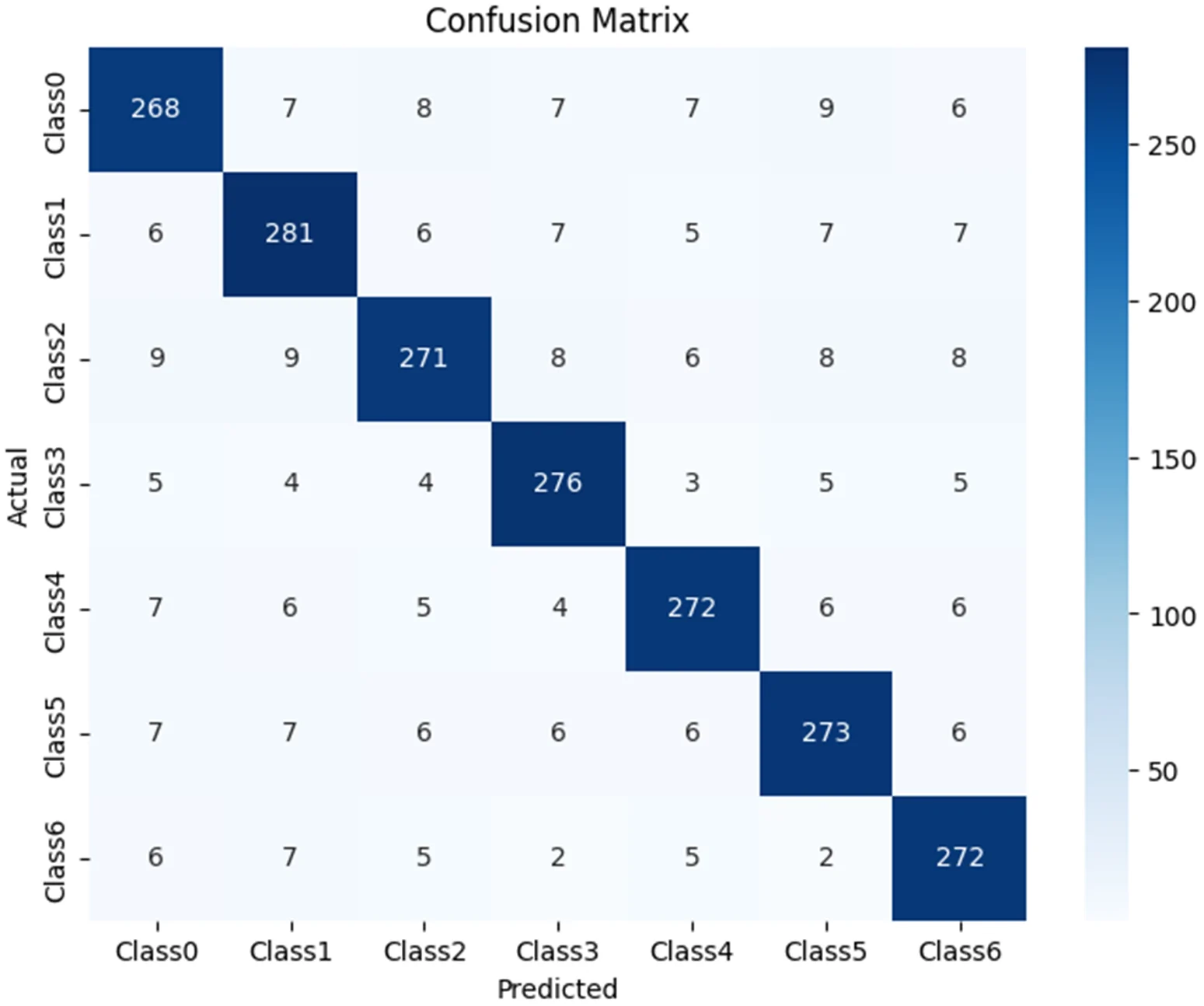
Confusion matrix of the LightGBM model.

**Fig 11:**
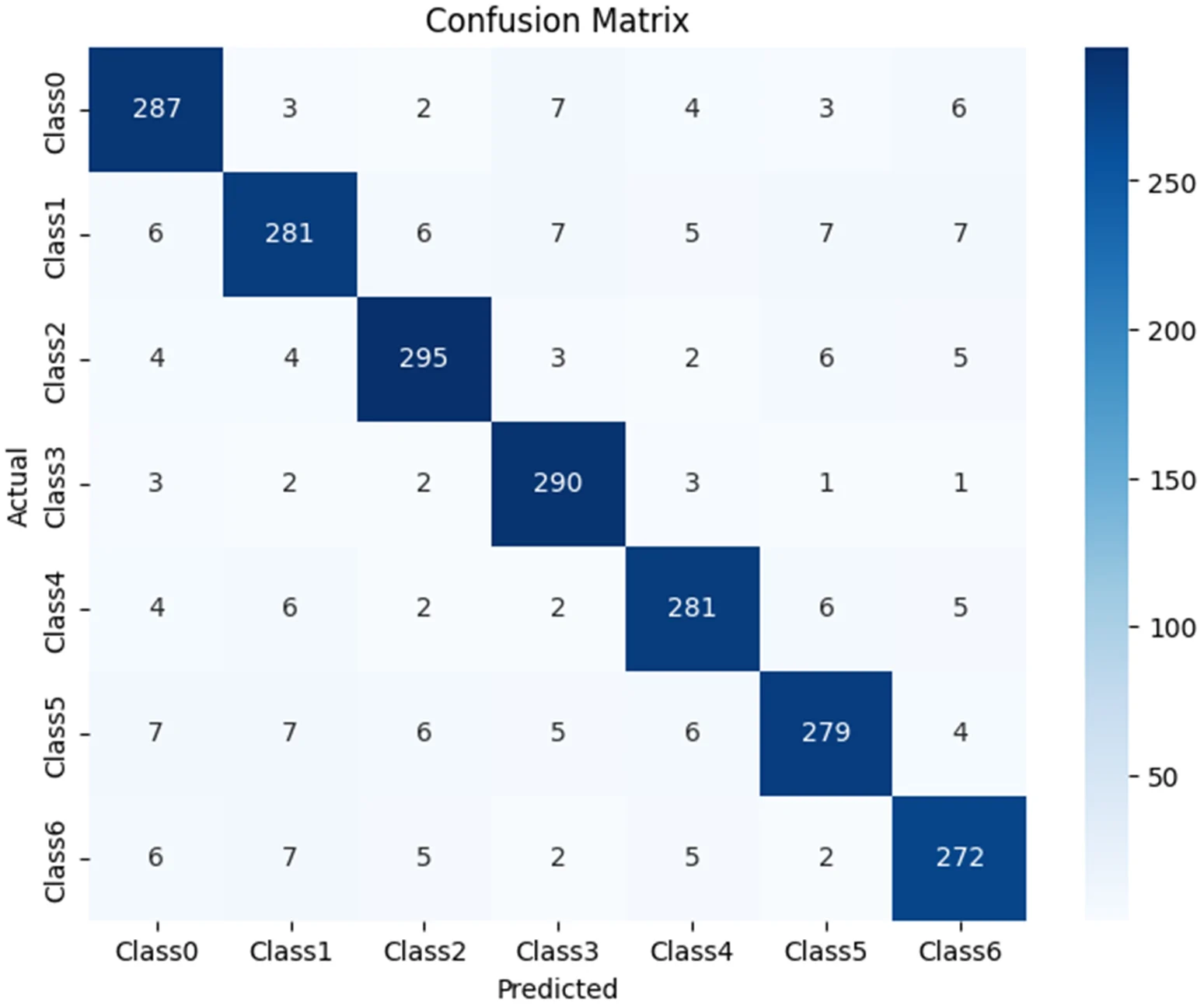
Confusion matrix of the XGBoost model.

**Fig 12:**
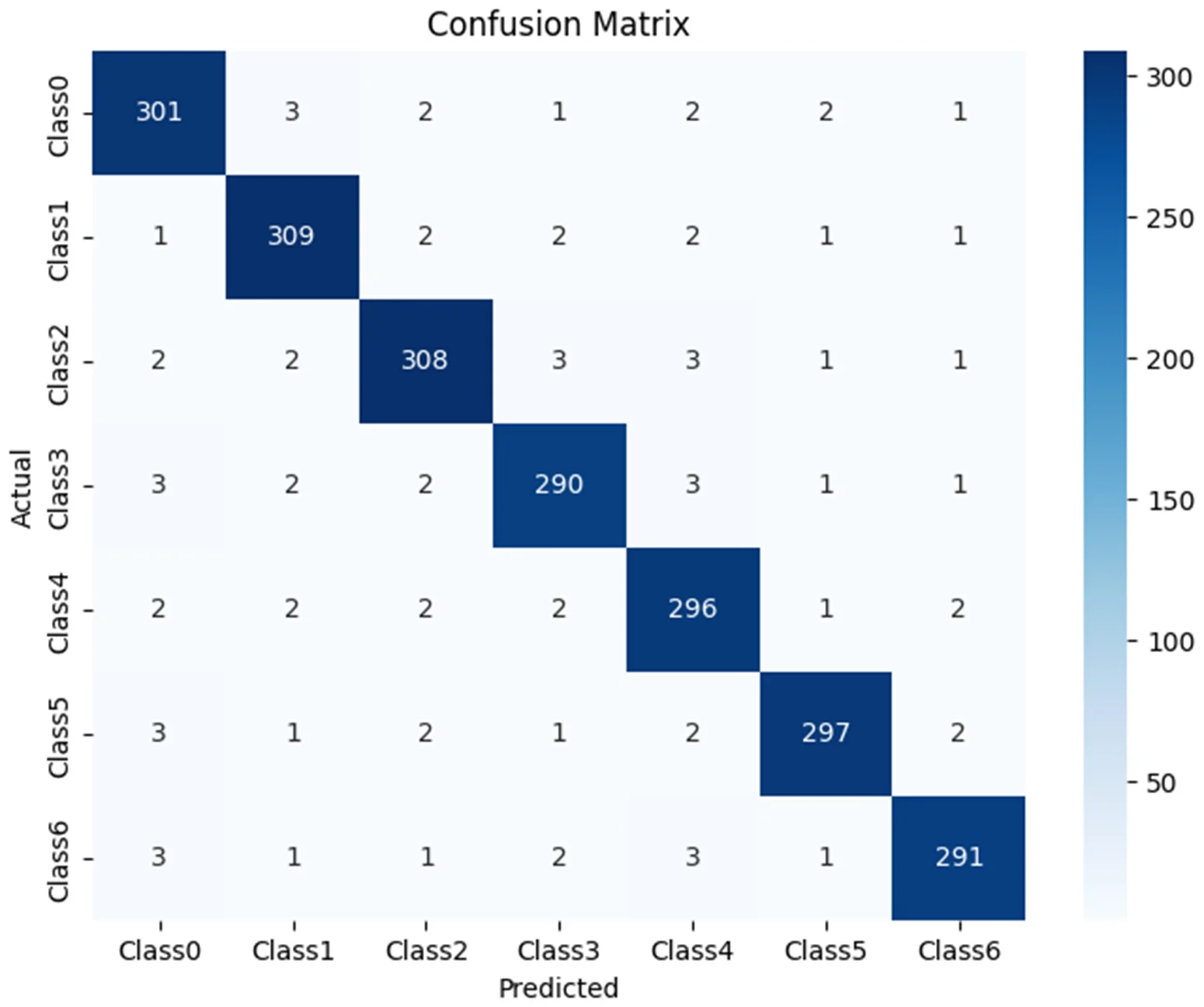
Confusion matrix of the MAX-voting model.

In summary, there are several major conclusions regarding the classification efficiency of various models. First, boosting classifiers (LightGBM and XGBoost) show improved accuracy compared to RF; second, MV ensemble model further improves the overall classification performance. The analysis of confusion matrices shows that RF classifier suffers from misclassification rates in Class1, Class3, and Class5; LightGBM partially eliminates these problems; XGBoost significantly reduces misclassifications, especially in Class2 and Class3; finally, MV further enhances differentiation by refining decision boundaries and combining several classifiers’ predictions. The analysis of error results through confusion matrices proves the superiority of ensemble methods over individual classifiers in terms of classifying skin lesions. In particular, the most accurate results can be obtained using MV classifier; XGBoost classifier delivers slightly worse but still high classification performance, whereas the lowest rates belong to RF. The transition from RF to LightGBM, then to XGBoost, and finally, to MV suggests that boosting methods and ensemble learning play an essential role in solving medical image classification problems, especially when the dataset is imbalanced. Thus, further efforts should be undertaken to optimize the model for improving classification efficiency, e.g., implementing hybrid stacking, tuning thresholds, etc.

### HAM10000 dataset: Cross-model performance analysis

This paper evaluates the performance of the models RF, LightGBM, XGBoost, and Majority Voting (MV) on the HAM10000 dataset in terms of accuracy, precision, recall, and F1-score. For the more precise estimation of model performance, 95% confidence intervals were calculated using the bootstrapping method with 1,000 resamples. The evaluation results can be found in Table 3. Among the individual models, XGBoost exhibits the best performance, whereas the MV ensemble yields the best results and achieves 95.94% accuracy.

**Table 3:** HAM10000 dataset: Cross-model performance with 95% confidence intervals.

Model	Accuracy (%)	Precision (%)	Recall (%)	F1-Score (%)
RF	85.76 (84.91–86.58)	84.50 (83.66–85.31)	86.00 (85.18–86.79)	85.24 (84.39–86.03)
LightGBM	88.16 (87.34–88.94)	87.10 (86.26–87.91)	88.50 (87.72–89.23)	87.79 (86.98–88.57)
XGBoost	91.47 (90.76–92.15)	90.80 (90.02–91.52)	91.90 (91.18–92.56)	91.34 (90.60–92.03)
MV	95.94 (95.28–96.49)	95.80 (95.16–96.37)	96.90 (96.31–97.40)	96.34 (95.72–96.88)

As follows from confidence intervals, MV does not become unstable during bootstrap resampling and demonstrates better performance compared to individual classifiers in terms of all considered criteria. This confirms that combining complementary models within an ensemble using majority voting increases predictive performance. This improvement is especially important for skin lesions since similar appearance of classes can lead to erroneous predictions.

Class-wise performance can be seen in Table 4, demonstrating consistency of the results obtained for all lesion types. Moreover, as shown in the table, narrow confidence intervals imply the stability of class-wise model performance during bootstrap resampling and not the result of a couple of lucky splits. The best results in terms of precision and F1-score are achieved for Class6 (VASC). On the other hand, Class5 (MEL) demonstrates relatively low recall values implying that melanoma detection remains the most difficult task among those examined. However, the difference between all other results remains minor, which means that the models exhibit generalization capabilities across classes. Figure 13 illustrates a Receiver Operating Characteristic (ROC) curve for three most dangerous skin cancer classes from the HAM10000 dataset. The ROC curve helps to evaluate classifier performance by plotting the True Positive Rate against the False Positive Rate.

**Table 4:** HAM10000 dataset: Class-specific results of the MV approach with 95% confidence intervals.

Class	Avg. Precision (%)	Avg. Recall (%)	Avg. F1-score (%)
Class0 (AK)	95.86 (95.12–96.42)	96.47 (95.74–97.03)	96.16 (95.43–96.72)
Class1 (BCC)	95.96 (95.24–96.53)	96.87 (96.15–97.31)	96.41 (95.68–96.93)
Class2 (BK)	96.55 (95.88–97.09)	96.55 (95.93–97.06)	96.55 (95.91–97.02)
Class3 (DF)	96.35 (95.61–96.91)	96.03 (95.29–96.67)	96.19 (95.47–96.79)
Class4 (NV)	96.10 (95.39–96.68)	96.73 (96.08–97.22)	96.42 (95.72–96.97)
Class5 (MEL)	96.74 (96.06–97.22)	95.81 (95.10–96.39)	96.27 (95.58–96.84)
Class6 (VASC)	97.32 (96.69–97.78)	96.36 (95.72–96.90)	96.84 (96.18–97.31)

**Fig 13:**
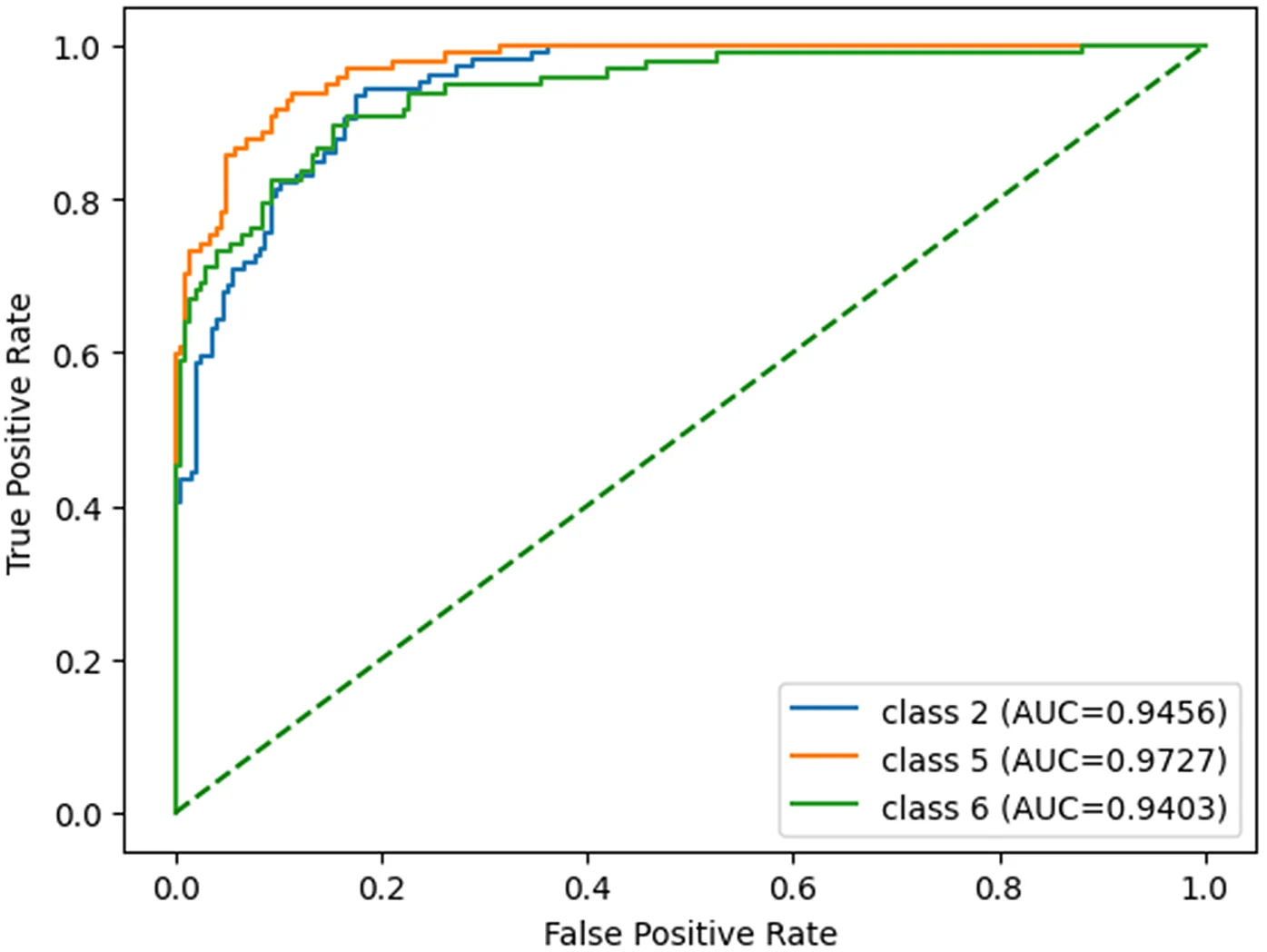
ROC curve of most dangerous types of skin cancer.

### Ablation study: Performance comparison

An ablation study is important for testing the influence of different elements in a machine learning algorithm. In our case, as can be seen in Table 5, we test the performance of several ML model configurations, with and without GA feature selection implementation. Feature optimization becomes crucial for increasing classification efficiency in this research. Initially, ML algorithms were tested without implementing GA feature selection. Random Forests produced 83.61% accuracy, whereas XGBoost and LightGBM provided even better results with 87.64% and 86.2% classification rates respectively. Ensemble learning helped to improve the performance of machine learning algorithms in case of no feature selection implementation. For example, Max Voting configuration with the implementation of Random Forest, XGBoost, and LightGBM provided an impressive 94.70% accuracy rate. Feature optimization was carried out with the help of GA, which helped increase model accuracy significantly. The best results were obtained by the ensemble of models with Max Voting implementation and GA, providing accuracy of 95.94%, as well as high recall (94.5%), precision (94.2%) and F1-score (94.35%). Feature selection ensured by GA reduced the number of features and made the classification less redundant. From the analysis of the ablation study, one can identify the following key observations:

- Without Genetic Algorithm: Max Voting model with RF, XGBoost, and LightGBM produced 94.70% accuracy rate.
- With Genetic Algorithm: Implementation of GA helped to optimize machine learning models significantly, resulting in 95.94% accuracy and higher results in terms of recall, precision, and F1-score for all tested algorithms.
- Ensemble model with Max Voting and GA provided more accurate results than the same model without genetic algorithm.
- Performance increase can be noticed not only for ensemble learning but also for individual machine learning models.

**Table 5:** Ablation study: Performance comparison of different model combinations.

Model Combination	Accuracy (%)	Recall (%)	Precision (%)	F1-Score (%)	Remarks
Random Forest (RF)	83.61	88.32	81.35	84.69	Base model without GA.
XGBoost	87.64	88.36	89.33	88.84	Base model without GA.
LightGBM	86.20	73.74	84.06	78.56	Base model without GA.
RF + XGBoost	89.12	91.00	87.00	88.50	Combining RF and XGBoost without GA.
RF + LightGBM	87.90	79.00	85.00	81.50	Combining RF and LightGBM without GA.
XGBoost + LightGBM	89.50	85.50	87.00	86.20	Combining XGBoost and LightGBM without GA.
Max Voting (RF + XGBoost + LightGBM)	94.70	93.23	92.54	92.88	Best performance without GA, full ensemble model.
Max Voting (RF + XGBoost + LightGBM) + GA	95.94	95.80	96.90	96.34	Best performance with GA for feature optimization, further improved accuracy and F1-score.
Max Voting (XGBoost + LightGBM) + GA	94.12	92.50	93.43	92.96	Max Voting with GA improves accuracy over the base model, but full ensemble with GA is better.
Max Voting (RF + XGBoost) + GA	94.12	93.00	92.10	92.55	Adding GA to the RF and XGBoost combination improves performance over the model without GA.

Thus, from the conducted ablation study, we may conclude that GA helps significantly to enhance the performance of machine learning algorithms, including their accuracy, F1-score, and other classification rates. The most effective model in terms of classification accuracy proved to be Max Voting with GA (95.94%), being thus optimal for precise skin cancer detection.

## Conclusion

This work demonstrates that the use of multiple machine learning models, together with a range of Explainable AI approaches, is capable of improving diagnostic accuracy while also meeting the transparency requirements necessary for successful adoption in healthcare settings. In particular, by combining Random Forest, XGBoost, and LightGBM through a Max Voting algorithm, alongside the use of Genetic Algorithms for feature selection, this model performs with near state-of-the-art accuracy on the challenging dermoscopy images. Importantly, the use of seven different approaches that fall under the Explainable AI paradigm, namely spatial methods such as Grad-CAM, pixel-wise explanation methods such as Integrated Gradients and DeepLIFT, smoothed explanations such as SmoothGrad, local explanations through LIME, and global approaches such as SHAP, provides interpretable output. This differs from traditional “black box” solutions, which do not offer clear explanations behind their decisions. Future work will consider practical applications to clinical settings, broader validation studies using various image modalities and diverse patient populations, and hybrid deep learning ensemble solutions to improve both accuracy and transparency.
